# Fathers’ Financial Support of Children in a Low Income Community in South Africa

**DOI:** 10.1007/s10834-013-9385-9

**Published:** 2014-01-21

**Authors:** Sangeetha Madhavan, Linda Richter, Shane Norris, Victoria Hosegood

**Affiliations:** 1 University of Maryland, College Park, MD USA; 2 Human Sciences Research Council, University of Kwazulu Natal, Johannesburg, South Africa; 3 University of the Witwatersrand, Johannesburg, South Africa; 4 University of Southampton, Southampton, UK

**Keywords:** Fathers, Financial support, Children, Kin, South Africa

## Abstract

We used data from the Birth to Twenty Cohort study to understand children’s receipt of financial support from their fathers in a low income, Black community in urban South Africa. Specifically, we (1) described fathers’ financial support over the life course of children; (2) estimated survival probabilities of *receiving support* for all children and *not* *receiving support* for children who experienced a parental union dissolution; and (3) identified factors that explained variation in the receipt of support after a union dissolution. Results suggest that most children received full or partial support throughout the life course. Furthermore, a high proportion of children received support after a union dissolution with much of the variation driven by pre-dissolution support, father’s education and the presence of extended kin.

## Introduction

A common portrayal of Black fathers in South Africa is that of the “deadbeat dad,” men who are unwilling to provide financially for their children. This image becomes even more prominent for fathers in the aftermath of a union dissolution with the child’s mother. However, in a context marked by high rates of unemployment and union instability, poor Black fathers struggle to meet their responsibilities as providers (Hunter 2007; Wilson 2006). Moreover, extended kin may influence the extent to which fathers provide financial support in the context of both an intact union and in the aftermath of a union dissolution. While research on fathers and fathering in the South African context has been growing (Hosegood and Madhavan 2013; Madhavan et al. 2008; Morrell and Richter 2006; Swartz and Bhana 2009), very little attention has been paid to the complexities of fathers’ financial support provision to their children (Hosegood and Madhavan 2010). To address this imbalance, we drew on data from the Birth to Twenty Cohort Study in Johannesburg, South Africa to (1) describe the extent of fathers’ financial support over the early life course of children; (2) estimate survival probabilities of *receiving support* for all children and estimate survival probabilities of *not* *receiving support* for those children who have experienced a parental union dissolution; and (3) identify factors that explain variation in the receipt of paternal financial support in the post-union dissolution context. Financial support in this analysis pertains to both state mandated and informal means of provision.

The value of the current analysis can be appreciated in several ways. One is the conceptualization of fathers’ roles as providers. Borne out of necessity and cultural pressures, the provision of financial support for children among Black men in a low-income context in South Africa needs to be examined as a fluid process amidst shifting configurations of care for children and adaptation over the life course. In this sense, the South African context offers a unique opportunity to advance understanding of how marginalized men exercise agency in meeting their fathering responsibilities under condition of pervasive social inequality. Secondly, the use of the Birth to Twenty (Bt20) dataset allowed us to use a life course perspective in studying father involvement. Thirdly, this analysis contributes to a growing trend to move away from hegemonic models of fathering based on white, middle class norms and consider alternative formulations of supportive fathering in low-income contexts (Cabrera et al. 2008; Myers 2013) and in non-Western contexts (Nsamenang 2010; Schwalb et al. 1987). Finally, the findings from this analysis make an important contribution to the study of low-income fathers globally and to policy development aimed at strengthening the role of fathers in promoting the well-being of children growing up in disadvantaged contexts.

## The South African Context

The challenges that Black men face in South Africa in relation to family life have been well documented. Whereas overall unemployment stood at 24 % in 2012, the unemployment rate for Black men was at 30 % (Statistics South Africa 2012). For Black fathers in South Africa, unemployment affects their ability to interact with their children in several ways. As they are expected play the provider role for their children (Moodie and Ndatshe 1994; Silberschmidt 1999), fathers who are not able to provide financially face shame and depression (Case and Wilson 2000; Thabane and Guy 1984) and are likely to disengage. The popular press is replete with testimonies from poor fathers who lament their inabilities to provide and, therefore, meet their responsibilities. The lack of local employment opportunities forces fathers to leave home to look for work (Wilson 2006) which inhibits direct communication with their children and fundamentally disrupts the established family structure (Moodie and Ndatshe 1994). Madhavan et al. (2008) showed, however, that non-coresident fathers were able to maintain contact with their children and provide financial support. Moreover, the children of fathers who were labour migrants have been shown to be mobile, themselves, suggesting an indirect effect of stable employment on fathers’ influence in their children’s lives (Bennett et al. 2014; Madhavan et al. 2012). The link between unemployment and union status is also important. Lack of or poor employment prospects limit men’s ability to pay bride price and afford marriage and serves as disincentives for Black women to enter into and/or remain in formal unions (Hunter 2009; Posel et al. 2011). Finally, even though South Africa has a long established legal framework for child maintenance payments by fathers in the aftermath of divorce, unemployed fathers are not forced to pay maintenance and penalties are not administered consistently (Burman and Berger 1988; Khunou 2012). However, fathers are increasingly challenging custody rulings over children that have traditionally favoured mothers (Gallinetti 2009; Khunou 2012), which, in turn, would have implications for fathers’ responsibility for providing financial support.

The inability to provide financially also fundamentally affects a father’s ability to meet other responsibilities. Fathers are expected to provide moral guidance and affection to children through communication, playing, companionship and role modelling. Not having a paternal link, made evident in not carrying a father’s surname or acquiring his clan name, is cause for great concern for children and youth (Ramphele 2002; Ramphele and Richter 2006) and their families (Madhavan 2010). Others have emphasized the unique contributions of fathers to their children such as in the provision of social capital, emotional support, and most importantly, love and care (Morrell 2006; Nsamenang 2000). Taken together, fathers have lost status in the domestic sphere (Lesenjane 2006), and this is compounded by negative portrayals of fathers as disengaged and irresponsible, particularly towards their children (Morrell and Richter 2006). Therefore, it is essential that we gain a better understanding of the dynamics of financial support provision using robust data.

## Determinants of Fathers’ Support Provision

Whereas the provision of financial support is seen as a universal expectation of fathers (Lamb 1997), there is variation in expectations and practice of how much and how often fathers provide (Coley and Chase-Lansdale 1999; Rangarajan and Gleeson 1998) which is also closely linked to employment trajectories in low income communities (Roy 2005). Financial support provision by fathers becomes even more challenging following the dissolution of a union when fathers do not co-reside with their children and may be in new relationships with other children, who either come with the women with whom they are in relationships or are born into the new unions. Research on child support payments in the US has examined the factors associated with assuming financial responsibility for children after divorce (Coleman and Ganong 1992). Other work has used social exchange theory to show that the provision of financial support to non-resident children after divorce was not cost effective because fathers did not benefit from their children’s attention and affection on a daily basis (Seltzer et al. 1998; Weiss and Willis 1985). Yet, fathers living apart from their children have been shown to continue to spend time and money on them (Hill et al. 2008; Hofferth and Anderson 2003; Madhavan et al. 2008). Therefore, it is important to better understand the sources of variation in fathers’ support provision following a union dissolution.

Our conceptual grounding for examining this issue has four critical dimensions that together explain why some fathers provide financial support while others do not in the period following a union dissolution.

### Paternal Attributes

Research on fathers’ age has shown that men who father children at a young age may not be financially ready to take on the responsibilities of fathering and in particular, providing financial support (Danziger and Radin 1990; Swartz and Bhana 2009). Not surprisingly, education and employment have been shown to positively impact the amount of economic support provided by fathers (Rangarajan and Gleeson 1998). Research on the effects of fathers’ remarriage on involvement with children from previous unions has supported Furstenburg’s “swapping families” hypothesis (1995), that is that fathers transfer financial investments to new co-resident biological children following a union dissolution (Hofferth et al. 2010; Manning and Smock 2000).

### Maternal Attributes

It has been well established that mothers play an important role in mediating the relationship between fathers and their children (Allen and Hawkins 1999; Amato and Gilbreth 1999). The extent to which mothers support or inhibit father involvement depends on, among other factors, age at the birth of the child, educational attainment and whether the mother enters a new relationship after union dissolution with the child’s father. Young mothers may lack the skills to manage the relationship between their children and the fathers, same or different, effectively. This may also hold true for those mothers who have minimal educational attainment (Lundberg et al. 2007). Mother’s influence on father involvement also depends on her entry into a new union which may influence father’s willingness to provide for his children. Research has shown that there is likely to be more ambiguity in how biological fathers relate to their children when step-fathers are involved (White and Gilbreth 2001). Moreover, Carlson and McLanahan (2004) have shown that the direction and quality of the relationship between parents is a critical predictor of father involvement post-dissolution. The presence of a new partner is likely to alter the nature of this relationship.

### Child Attributes

Some research has shown that “closeness” to biological mothers and fathers declines as children age (Heatherington and Clingempeel 1992; Hofferth 1998). This phenomenon does not appear to be reflected in child support patterns which have shown that older children are more likely to receive child support than younger children (Furstenberg and Harris 1992; Seltzer 1991). Indeed, a broader developmental perspective has been emphasized as necessary to understand how father involvement responds to shifts in children’s age-related development needs (Palkovitz and Palm 2009; Parke 2000). Child’s sex has been shown to have an effect on overall levels of father involvement usually favouring boys (Harris and Morgan 1991; Lamb et al. 1987), particularly among unmarried couples (Lundberg et al. 2007), but has been shown to have no direct effect on father’s provision of financial support (Lundberg et al. 2007).

### Role of Kin

Far less attention has been paid to the role of kin in influencing the provision of financial support by fathers. There is a well-established line of research in Africa that has demonstrated that the biological relationship between fathers and their children needs to be situated within a larger web of relationships with kin (Lesenjane 2006; Riesman 1992; Townsend 2000) and that kin play an important role in child rearing (Mkhize 2004, 2006). For example, in many Black communities in southern Africa, the oldest brother of an unmarried woman with a child has been known to have key paternal responsibilities on behalf of the mother’s family and would essentially function as a “social father” (Junod 1962; Niehaus 1994). This may include the provision of financial support, moral guidance and practical assistance for school and other activities. Madhavan and Roy (2012) have shown how the practice of “kinwork”—the work that various kin members do to keep families functioning and to rear children—operates to support fathering in low income Black communities in South Africa and the US. In the US context, it has also been shown that mothers actively recruit “social fathers” from their kin networks to help with childrearing (Roy and Burton 2007). The role of kin is so important that, even where child support is mandated by law, women are reluctant to use it in favour of kin support. Garey and Townsend (1996) have argued that, in Botswana, few women actually use the child support mechanism because it interferes with traditional mechanisms of support for their children (i.e., extended kin) and can jeopardize the women’s chances of eventual marriage with the biological fathers or another man.

However, the influence of extended kin can sometimes be contentious, particularly when unions are not formalized or in the post-union dissolution context. In their study of fathers in Cape Town, South Africa, Swartz and Bhana (2009) have described how extended kin both facilitate and inhibit young fathers from developing relationships with their children when they are not in a formal unions with the mothers of the children. Kin, through their role as “gatekeepers” of children, may restrict fathers’ access to children after the dissolution of a union, particularly if the dissolution occurred under acrimonious circumstances, as has been demonstrated in Stack’s (1975) ethnography on “kin work” in a low income Black community in the US. Moreover, in contexts with high unemployment and scarcity of resources, kin may be wary of allowing non-resident fathers access to children for fear that the existing limited resources are further diluted. US-based research that has examined this relationship quantitatively has found either no effect (Danziger and Radin 1990) or an inhibitive one (Kalil et al. 2005). It is possible that fathers respond to kin gatekeeping by providing financial support to children as a means to ensure their roles in their children’s lives post-dissolution. On the other hand, fathers may respond in an opposite manner by withdrawing financial support in the face of kin gatekeeping. This is a key question that we addressed in our analysis.

The four dimensions discussed above can be applied to the South African context to better understand (1) the extent to which children receive financial support from fathers through the early life course, and more specifically (2) to identify the determinants of support receipt in the period following a parental union dissolution—two issues that have not been adequately addressed in the literature on fathering in South Africa. Specifically, we addressed the following research questions pertaining to each dimension:

1. Paternal Attributes: How do father’s age at birth and educational attainment influence children’s receipt of financial support following union?
2. Maternal Attributes: How do mother’s age at birth, educational attainment and entry into a new union influence children’s receipt of financial support following union?
3. Child Attributes: How does child’s sex and age at time of parental union dissolution influence children’s receipt of financial support following union?
4. Kin Involvement: How does the presence of non-parental adults in the household influence children’s receipt of financial support following union?

## Data and Methods

### Data Description

Bt20 has been the longest running birth cohort study in Africa situated in the greater Johannesburg-Soweto municipality in South Africa (Sabet et al. 2009; Yach et al. 1991). The majority of families, most of whom were Black, came from socioeconomically disadvantaged circumstances. Bt20 was initiated as an observational, systematic study of human development, health and well-being, from birth extended through to young adulthood. Data collection covered a broad range of topics including anthropometric measures, nutrition, family composition, socioeconomic circumstances, childcare, parenting, cognitive development, and social experiences at home, school, and in the community. Prospective data collection began in the antenatal period and continued with approximately 21 follow up visits until age 23. Children born between April and June 1990 and resident for at least 6 months in the Soweto-Johannesburg municipality were enrolled into the study (*n* = 3273). The cohort included Black, White, Indian, and Colored children but we limited this analysis to only the Black children (*n* = 2568) who comprised the largest proportion of the cohort in line with the population distribution of the area. Even though data have been collected through age 23, this analysis used age 18 as the end point. While these data are not nationally representative, they offer some of the richest data on father involvement in the South African context, and therefore, are highly suitable for this analysis.

#### Data on Fathers

Data in Bt20 on father involvement have been collected in two ways. Prospective data collected as part of household rosters to determine father co-residence, father contact and provision of financial support by fathers for most years of data collection. In addition, a retrospective questionnaire specifically focusing on father involvement across the child’s life course was administered at year 18. The questionnaires, most of which were answered by mothers, included detailed information on fathers’ co-residence with the child, extent of contact if not co-resident, provision of financial support, and other forms of interaction with the child for every year from birth until age 18. To both maximize our sample size and improve the validity of our measures, we used the retrospective data to supplement the prospective data but always privileged the prospective when it was available. There are two drawbacks that need to be acknowledged. One, most of the information about fathers came from mothers or other caregivers. Research from the US context has highlighted the potential biases in mothers’ reports, which consistently show underreporting of father involvement (Coley and Morris 2002). It is difficult to establish the extent of such bias in the Bt20 data but comparison of mothers’ reports of father contact over the life course and fathers’ reports of their own involvement (from the year 18 biological father questionnaires) suggested potential underreporting. Two, the use of retrospective data introduced problems associated with memory recall the farther back in time that data were sought. However, when we compared retrospective reports of father presence in the 0–2 year period with prospective data for the same time period, we found that 85 % of reports matched.

### Analytical Sample

Attrition over the course of the BT20 study has been about 30 %, mostly occurring during infancy and early childhood when women moved back to their rural homes after giving birth (Norris et al. 2007). A small number of children were lost to follow-up as a result of death. There have been very few withdrawals from the study. After removing non-Black children, the sample was 1,942 girls and boys followed up from birth to age 18, out of which, 1,557 were administered the retrospective father questionnaire. Table 1 shows descriptive characteristics of the analytical sample at the time of birth.

**Table 1:** Selected characteristics of analytical sample at time of birth (*N* = 1,557)

Sex of child		Paternal education	
Male	48.6 %	No schooling	0.3 %
Female	51.4 %	Some primary	2.0 %
Parity		Completed primary	4.2 %
1	38.2 %	Some secondary	21.7 %
2	29.3 %	Completed matric	30.1 %
3	17.0 %	Post-school	11.6 %
4+	15.5 %	Missing	30.1 %
Maternal age (mean)	25.8	Household wealth index	
Mother’s marital status		1	15.0 %
Married	29.7 %	2	17.4 %
Living together	3.9 %	3	31.9 %
Divorced/widowed	1.0 %	4	18.8 %
Single	65.1 %	5	9.4 %
Missing	0.3 %	Missing	7.6 %
Maternal education			
No schooling	0.8 %	Household structure	
Some primary	5.3 %	Nuclear family	19.5 %
Completed primary	6.7 %	Extended family	63.3 %
Some secondary	41.8 %	Missing	17.2 %
Completed matric	31.9 %		
Post-school	6.9 %		
Missing	6.6 %		
*N*	1,557	*N*	1,557

A little more than a third of the cohort was composed of first births and the mean age of mothers at birth of the index child was 25.8 years. More than a third of all mothers were married or living together (a term used synonymously with cohabiting) with their partners. The majority of mothers had at least some secondary school education. We found a similar distribution for fathers on educational attainment though there was a sizeable missing proportion. The household wealth index used in this analysis was computed as quintile rankings of asset scores based on home ownership, access to regular electricity and ownership of car, TV, refrigerator, and phone. It ranged from 1 (very poor) to 5 (wealthy) and showed the highest proportions in the 2nd and 3rd quintile. Finally, the majority of households were classified as extended family structure although there were a sizeable number of records with missing data.

### Analytical Method

Children’s receipt of financial support was treated as a dichotomous outcome (1/0) based on responses to the question, “In the past year, who was mainly responsible for the material support of the child?” To examine the timing of children receiving financial support from fathers, we used Kaplan–Meier estimation techniques to determine the survival probabilities of (1) receiving financial support from fathers for all children, and (2) not receiving financial support from fathers for those children who experienced a union dissolution. Although we recognized that the events of interest could recur, in these analyses we considered only the first observed event because of insufficient sample size.

To examine correlates of father support provision post-union dissolution, we used a discrete time event history model. The child cohort was comprised of all children who had ever experienced a parental union dissolution before the age of 18. A child entered the cohort at the year of parental union dissolution. The dependent variable or event of interest occurred when a child received financial support from the father for the first time post-dissolution. Children who received support in the year of dissolution were included in the analysis and their odds of experiencing the event started at the year of dissolution. An observation was censored if the event did not occur by the age of 18 when the observation period ended or when the father died. Each child’s exposure time was divided into child-years starting at the time of parental union dissolution and consisting of 1 year intervals resulting in 3,777 child years of exposure. We used logistic models in SPSS to estimate the odds of children receiving financial support in the post-dissolution period.

Paternal attributes included father’s age and educational level at time of birth of the child, which was also treated as a measure for employment potential. The maternal characteristics included mother’s age and education at time of birth, and whether she entered a new union following dissolution in the first 5 years after dissolution. Child characteristics included sex of child, and age at time of parental union dissolution categorized into four developmental stages (0–2, 3–5, 6–11 and 12–18) and entered as a continuous variable. Kin involvement was treated as a continuous variable measured by number of co-resident non-parental adults (data available in the household rosters). Control variables included household wealth at time of birth measured by quintile ranking of asset score based on ownership of household items (1–5) and whether father provided any support before or at the time of union dissolution. All covariates were time constant except number of co-resident non-parental adults which was treated as time varying and measured at the beginning of each period.

#### Survival Bias

In our quest for maximizing sample size by integrating retrospective data with the prospective data, the analytic sample was composed of only those children who “survived” in the study until year 18. It is, therefore, possible that those children who were lost to follow up might have had weaker links to their fathers which would, in turn, contribute to an overestimation of father involvement in our analysis. We examined this issue by comparing the means of duration of father contact for children who dropped out and those who did not by age of attrition. With the exception of two attrition periods: 6 months–2 years and 12–13, none of the differences were significant suggesting that our estimates of father contact in this analysis were not seriously affected by survivor bias.

## Results

Table 2 shows the proportion of children in selected support receipt types across 5 year age groups for all children. Children whose fathers died at some point in the period were treated as a separate category and not included in the denominators of the other proportions.

**Table 2:** Proportion of children receiving different types of support across age groups

	0–5	5–10	10–15	15–18
Receives uninterrupted financial support from father in period	61.5 % (958)	53.4 % (831)	44.7 % (696)	38.8 % (604)
Receives partial financial support from father in period	10.5 % (163)	19.5 % (303)	26.6 % (414)	12.6 % (196)
Receives no financial support from father in period	23.6 % (368)	23.2 % (361)	20.4 % (318)	31.9 % (496)
Dead fathers	0.8 % (12)	3.9 % (60)	8.3 % (129)	16.8 % (261)
Total	1,557	1,557	1,557	1,557

*Notes* Dead fathers removed from denominator of all percentages. Cumulative percentages across life stages. Age groupings are not inclusive of the endpoint year with the exception of the oldest age group which is truncated at age 18. Percentage missing ranges from 0 to 4 % across years

**Fig. 1:**
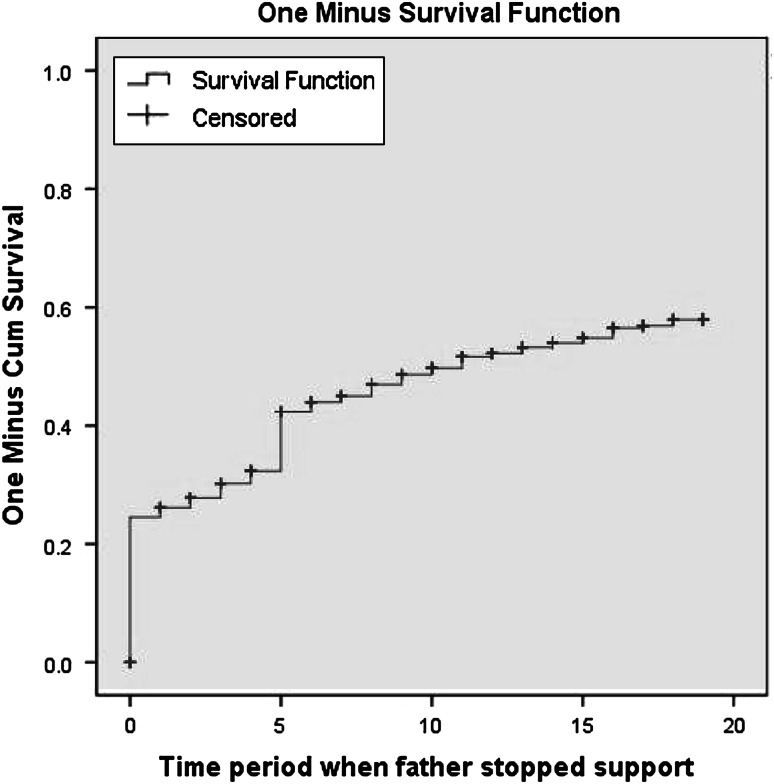
First experience of not receiving financial support for all children (*N* = 1,557)

The proportion of children who received uninterrupted financial support from their fathers decreased from a high of 61.5 % at ages 0–2 to below 38.8 % for the oldest age group. The proportions who received partial and no support during the period increased across age groups. The decrease in partial support found in the oldest age group was attributable to having fewer years in the last interval, which, in turn, resulted in a shorter exposure time in which fluctuations could occur. We now turn to Kaplan–Meier survival functions to gain a better understanding of the timing of support provided by fathers in children’s lives. The curve in Fig. 1 shows the one minus survival function for experiencing a first event of “not receiving financial support from fathers” for all children.

Twenty percent of children started out life not receiving financial support from their fathers. The percentage of children who experienced a first event of no support increased to 45 % by age 5 with a further gradual increase to 55 % by age 18. We know that much of this increase was attributable to parental union dissolution (analyses not shown). What we do not know is how many children received financial support after union dissolution, even if they were not receiving it at the time of dissolution, and what accounted for the variation in receipt of financial support in this context. This is what we examined in the remainder of the analysis.

**Fig. 2:**
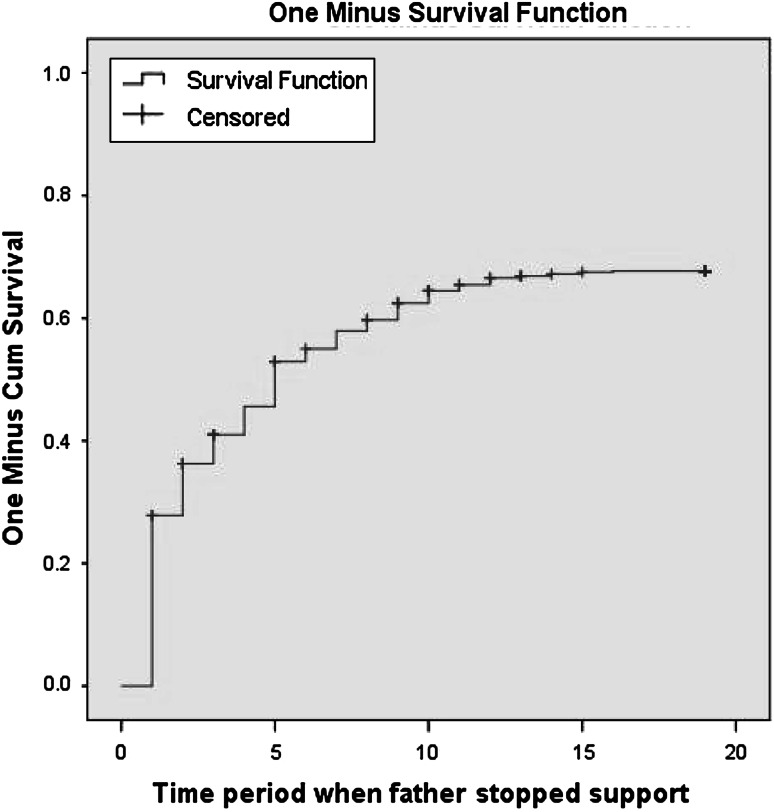
First experience of receiving financial support after union dissolution (*N* = 690)

Out of the 690 children who experienced a union dissolution at some point in their lives, 457 were not receiving support at the time of dissolution. Figure 2 shows the one minus survival function for first experience of receiving financial support from fathers after a union dissolution.

We found that 30 % of children received financial support within a year of dissolution. The majority of these children were receiving support from their fathers before and at the time of dissolution. A further 25 % received support within 5 years of dissolution and a full 65 % of children received support for the first time by the age of 18. Table 3 presents the results from four discrete time event history models predicting the odds of children receiving paternal financial support for the first time following union dissolution. Model 1 includes only paternal attributes with controls; Model 2 adds maternal attributes; Model 3 includes child attributes and the full Model (4) adds kin involvement.

**Table 3:** Odds of children receiving financial support from fathers post parental union dissolution

	Model 1	Model 2	Model 3	Model 4
	Odds ratio (SE)	Odds ratio (SE)	Odds ratio (SE)	Odds ratio (SE)
Paternal characteristics				
Age at birth of child	0.992 (0.011)	1.032 (0.017)	1.033* (0.017)	1.033* (0.017)
Education at birth of child	1.485*** (0.112)	1.495*** (0.115)	1.472*** (0.116)	1.493*** (0.117)
Maternal characteristics				
Age at birth of child		0.958** (0.015)	0.958** (0.015)	0.954** (0.015)
Education at birth of child		0.959 (0.120)	0.972 (0.120)	0.943 (0.121)
Entered new union		0.982 (0.116)	0.002 (0.116)	0.976 (0.116)
Child characteristics				
Sex of child (ref: female)			1.102 (0.110)	1.121 (0.110)
Age at time of union dissolution			0.957 (0.075)	0.956 (0.075)
Kin involvement				
Number of non-parental adults in household				0.945* (0.075)
Household wealth index	0.926 (0.047)	0.914 (0.050)	0.915 (0.050)	0.918 (0.050)
Pre-dissolution support provision	2.171*** (0.120)	2.205*** (0.122)	2.291*** (0.137)	2.291*** (0.137)
Nagelkerke R-square	0.035	0.039	0.040	0.042
*N*	3,392	3,392	3,392	3,392

*** Significant at the 0.001 level

** Significant at the 0.01 level

* Significant at the 0.05 level

Father’s educational level at the time of birth had a consistent strong positive effect on the odds of children receiving financial support even after controlling for pre-dissolution support which, itself, had the expected positive effect in all models. This is consistent with expectations that education, which was also being treated as a proxy for employment potential, was the biggest factor in predicting the receipt of paternal financial support. Father’s age had a weak positive effect in Models 3 and 4. Interestingly, mother’s age at the time of birth had a consistently strong negative effect on the odds of children receiving paternal support (although the reduction itself was fairly small). This is puzzling given that older mothers were expected to have more power to encourage or pressure their former partners to meet financial responsibilities. It is possible that these women may want to be more independent of their former spouses. We found no effect of mother’s entry into new union on their children’s receipt of paternal financial support. Unfortunately, we did not have data on relationship quality between fathers and mothers who were former partners, but this result suggests that fathers’ relationship to their non-resident children was somewhat independent of their mothers’ relationship trajectory and, by extension, the parental relationship. Finally, the number of non-parental adults in the household appeared to inhibit children’s receipt of financial support from their fathers though the significance was not very strong.

## Discussion

In this analysis, we studied fathers’ financial provision to their children over the course of childhood and the determinants of support following a parental union dissolution in an urban context in South Africa. Several key findings merit some consideration. Despite pervasive unemployment that makes it difficult for fathers to play the provider role for their children, a high proportion of children received either continuous or interrupted support from birth to age 18. However, there was variation in financial support receipt across age groups within the early life course. We found that nearly 40 % of children experienced a first event of not receiving support from their fathers in the first 5 years of life. This is similar to findings in the US literature that has documented the waning of father involvement in early childhood (Cutrona et al. 1998; Furstenberg and Harris 1993). Indeed, the proportion of children receiving uninterrupted support declined with age of the child reflecting the volatility of men’s employment, the effect of union dissolution and life stage of the child, e.g., it may be easier to pay for early childcare needs (diapers, food) than for school related expenses later on in life. Moreover, fathers may sense greater social approbation when they do not provide for the mothers of their very young children, an attitude that might not be as strong for fathers of older children.

Union dissolution which results in fathers moving out of the household (almost all couples in union were cohabiting in our sample) is undoubtedly a critical factor in understanding receipt of financial support from fathers. Fathers may not feel obligated to provide support once they move out of the house. Moreover, fathers may have to manage competing demands that emerge from subsequent childbearing with new partners who are able to make more demands than previous partners. However, our analysis showed that a large percentage of children did receive some financial support from their fathers following dissolution. The most significant determinants of receiving support post-dissolution were whether father provided support before the dissolution and father’s education, both of which increased the odds of a child receiving support; mother’s age and the presence of kin in the household had the opposite effect. Whereas the effect of “support provision before the dissolution” was expected, it is, nonetheless, notable because it suggested that fathers’ commitment to their biological children does not end when their unions to the mothers end. The effect of kin involvement is intriguing. It is possible that having access to kin who provide financial support may be a disincentive for fathers to provide financial support to their children. Unlike the US context where studies have shown that kin do not provide financial support to single mothers in the Black community (Hofferth 1984; Raley 1995), it is not uncommon for single women to not only live with their extended kin but to also receive support from them in South Africa. While it was difficult to tell whether fathers were getting “pushed out” by kin influence or whether fathers “disengaged,” it was evident that the role of kin was critical in understanding father involvement. In this sense, our findings are an extension of research in the US that highlights the role of “maternal gatekeeping” (Jarrett et al. 2002) in excluding fathers. In our analysis, it appeared that “kin gatekeeping” may be a critical factor as well in fathers’ involvement with their non-residential children. More work, particularly qualitative, is needed to fully understand these processes.

In interpreting these results, it is important to recognize limitations of this study. First, we restricted this analysis to the first event of either “not receiving” or “receiving” support. In a context in which union dynamics are volatile and where the connection to the formal labour market is tenuous at best for Black men, fathers’ provision of financial support is best understood as a process. Therefore, future analysis should model the receipt of financial support as a trajectory using appropriate statistical techniques that allow for modelling recurring events. Moreover, recent work suggests that the time span of examining father involvement, in any form, should be expanded to include the prenatal period (Shannon et al. 2009). The Bt20 dataset does include some data on prenatal conditions and, therefore, may enable such analyses. Second, is the measurement of covariates, many of which we have constrained to be time constant in this analysis. While conditions at the time of birth may have a unique effect on the timing of events later on in life, it is important to account for the time varying nature of these covariates, in particular, employment status. Future research should also examine the role of mothers in mediating father involvement using other measures. For example, maternal gatekeeping, through which mothers monitor the time that fathers spend with their children, and the quality of that interaction can be examined further by including more nuanced indicators of maternal contact and the amount of support that mothers provide. We also know that the quality of the parental relationship is a key determinant of father involvement (Carlson and McLanahan 2004). Moreover, disputes over financial expenditures are often the source of relationship conflict (Britt and Huston 2012) which introduces possible endogeneity issues in modelling fathers’ financial support in the context of union dissolution. Therefore, future work should incorporate measures of relationship quality including the extent of financial stability in modelling these effects. Finally, we did not examine the effect of fathers’ financial support on children’s well-being which, in the US context, has not shown the strong positive impact that might have been expected (Garasky and Stewart 2007; Hofferth and Pinzon 2011).

The findings from this analysis are important for a number of reasons. First and foremost, they support a growing body of evidence in the US and elsewhere that non-resident fathers continue to remain engaged with their children from a dissolved union. However, employment instability, which is linked to union instability, means that the support is likely to be inconsistent. Second, they underscore the importance of longitudinal data which are essential to demonstrate the influence of changing events over the life course. Third, our findings contribute to the ongoing discussions about applying white, middle class models of fathering to non-European, low-income populations where a combination of economic necessity and cultural preferences bring about markedly different models of parenting. Even using the limited purview of financial support and within that, a simplistic dichotomous indicator as we did in this analysis, it is clear that fathers’ ability to provide support to their children is constantly tested throughout the life course. Therefore, any conceptual model or empirical work on father involvement in such contexts must be able to reflect such exigencies. Otherwise we risk, at best, misunderstanding fathers’ roles in their children’s lives and, at worst, underestimating their actual contribution. Finally, our results also shed light on the complex role of extended kin in mediating the relationship of fathers and their non-resident children. Specifically, they underscore the need to use a more nuanced approach that goes beyond answering the question, “are kin supportive or not of fathers’ attempts to being involved with their children?” and considers dynamic models of family structure that both reflect and respond to life course needs of children and adults and the larger socioeconomic context in which parenting takes place.

The value of this analysis can also be appreciated as it relates to policy discussions about strengthening the role of fathers in their children’s lives, particularly in low income communities, in South Africa and elsewhere. Legal measures to force fathers to pay child support are increasingly utilized by women in South Africa (Khunou 2012) but are difficult to justify as long as unemployment remains high. However, it may be useful to consider modifications to existing programs and targeted interventions at critical junctures. The South African government provides a range of social grants to alleviate hardships faced by low income families. At present, child support grants are given to primary caregivers regardless of fathers’ survival status or contact. Rarely is there a concerted effort to even identity fathers, let alone engage them in the process, because it is assumed that most non-resident fathers are not involved with their children. Our findings underscore the need to redouble efforts to bring in fathers into any decision making involving their children. Most family and parenting support programs in South Africa offer a package of services including some form of financial assistance for families (Comer and Fraser 1998; Layzer et al. 2001). Financial assistance could be expanded to specifically assist or support men to manage their own resources in ways that allow them to provide financial support for their children (e.g., loan schemes that provide cash for men at the beginning of the school year or tax free savings for education and health costs). Given that fathers who have a history of providing financial support continue to do so after union dissolution suggests that fathers do not simply walk away from their children as commonly portrayed. Therefore, strengthening the message that fathers can develop a nurturing relationship with their children independent of their relationship with mothers and providing targeted services may provide the needed push to keep providing financial support. The fact that we did not find a negative effect of mothers’ entry into a new union should provide further impetus to this approach. Finally, the possible obstructive role of kin in promoting the provision of financial support by fathers in the post-dissolution context should invite careful consideration of intervention models that incorporate other family members. There are clearly others who have a vested interest in the welfare of children and therefore, should be incorporated into intervention programs. Finally, our findings have important implications for strengthening efforts to track father involvement in children’s lives, including their roles as providers (Sherr and Barry 2004) through better data collection. Taken together, these improvements to intervention programs and policies should reveal the critical role of fathers in child development which, thus far, has been under appreciated.
